# Using Google Glass in Surgical Settings: Systematic Review

**DOI:** 10.2196/mhealth.9409

**Published:** 2018-03-06

**Authors:** Nancy J Wei, Bryn Dougherty, Aundria Myers, Sherif M Badawy

**Affiliations:** ^1^ Weinberg College of Arts and Sciences Northwestern University Evanston, IL United States; ^2^ Division of Hematology, Oncology and Stem Cell Transplant Ann & Robert H Lurie Children's Hospital of Chicago Chicago, IL United States; ^3^ Department of Pediatrics Feinberg School of Medicine Northwestern University Chicago, IL United States; ^4^ Department of Pediatrics Division of Hematology and Oncology Faculty of Medicine, Zagazig University Zagazig Egypt

**Keywords:** Google Glass, wearable, wearable device, head-mounted wearable device, surgery, surgical setting, surgical condition

## Abstract

**Background:**

In recent years, wearable devices have become increasingly attractive and the health care industry has been especially drawn to Google Glass because of its ability to serve as a head-mounted wearable device. The use of Google Glass in surgical settings is of particular interest due to the hands-free device potential to streamline workflow and maintain sterile conditions in an operating room environment.

**Objective:**

The aim is to conduct a systematic evaluation of the literature on the feasibility and acceptability of using Google Glass in surgical settings and to assess the potential benefits and limitations of its application.

**Methods:**

The literature was searched for articles published between January 2013 and May 2017. The search included the following databases: PubMed MEDLINE, Embase, Cumulative Index to Nursing and Allied Health Literature, PsycINFO (EBSCO), and IEEE Xplore. Two reviewers independently screened titles and abstracts and assessed full-text articles. Original research articles that evaluated the feasibility, usability, or acceptability of using Google Glass in surgical settings were included. This review was completed following the Preferred Reporting Results of Systematic Reviews and Meta-Analyses guidelines.

**Results:**

Of the 520 records obtained, 31 met all predefined criteria and were included in this review. Google Glass was used in various surgical specialties. Most studies were in the United States (23/31, 74%) and all were conducted in hospital settings: 29 in adult hospitals (29/31, 94%) and two in children’s hospitals (2/31, 7%). Sample sizes of participants who wore Google Glass ranged from 1 to 40. Of the 31 studies, 25 (81%) were conducted under real-time conditions or actual clinical care settings, whereas the other six (19%) were conducted under simulated environment. Twenty-six studies were pilot or feasibility studies (84%), three were case studies (10%), and two were randomized controlled trials (6%). The majority of studies examined the potential use of Google Glass as an intraoperative intervention (27/31, 87%), whereas others observed its potential use in preoperative (4/31, 13%) and postoperative settings (5/31, 16%). Google Glass was utilized as a videography and photography device (21/31, 68%), a vital sign monitor (6/31, 19%), a surgical navigation display (5/31, 16%), and as a videoconferencing tool to communicate with remote surgeons intraoperatively (5/31, 16%). Most studies reported moderate or high acceptability of using Google Glass in surgical settings. The main reported limitations of using Google Glass utilization were short battery life (8/31, 26%) and difficulty with hands-free features (5/31, 16%).

**Conclusions:**

There are promising feasibility and usability data of using Google Glass in surgical settings with particular benefits for surgical education and training. Despite existing technical limitations, Google Glass was generally well received and several studies in surgical settings acknowledged its potential for training, consultation, patient monitoring, and audiovisual recording.

## Introduction

Wearable technology is defined as a compact device worn on the body as an implant or accessory that aids an individual’s activities without interfering with the user’s movements [1]. The goal of these technologies is to promote convenience and productivity by allowing the user to operate the device through voice and motion commands, thus offering more frequent and proficient multitasking opportunities. Many of these devices also possess the ability to connect to the Internet; therefore, they are capable of fulfilling the same functionality as mobile phones or computers [2]. However, wearable devices retain the added benefits of sustained hands-free portability and real-time ubiquitous access to data [3] compared with mobile phones or computers. One of the most well-known wearable devices is Google Glass (Google Inc, Mountain View, CA, USA), commonly referred to as “Glass,” which is an optical head-mounted display worn as a pair of spectacles.

First released as the Google Glass Explorer Edition in 2013, Google Glass emerged as a head-mounted device that employs a wireless interface designed to provide its users with a comfortable, multifunctional virtual or augmented reality experience [4]. Drawing from its Android operating system, Google Glass projects information onto a small screen positioned just above and to the right of the user’s right eye, creating little obstruction to his or her line of vision [5]. Google Glass offers a gateway for uninterrupted, instant information accessibility. Although the original Explorer Edition was unable to fully meet the needs of the general consumer population, its voice activation and data transmission capabilities, built-in camera, and flexibility of app customization has garnered the interest of commercial industries and professional operations, including health care [6].

In the health care industry, Google Glass has been used in different settings, including surgical and nonsurgical ones. In nonsurgical settings, Google Glass has been used to help clinicians in providing medical care for patients, health monitoring, and treatment plan support. For example, in patient-centered studies, researchers tested the role of Google Glass in helping colorblind patients identify colors and in providing audiovisual feedback to patients with Parkinson disease to modulate gait [7,8]. Further, as a clinician-centered intervention, Google Glass has been harnessed by health care providers to record medical consultations and to allow remote collaboration between physicians [9,10].

Recently, Google Glass’s multitasking capabilities and responsiveness to hands-free voice and motion commands have made it particularly attractive to the surgical field. These advantages present surgeons with the opportunity to better streamline workflow in a setting where maintaining sterile conditions in the operating room and continuously monitoring patients during surgery are crucial. Although the multifaceted capabilities of Google Glass offer the potential to greatly impact the surgical field, health care providers remain uncertain about which tasks can benefit most from Google Glass intervention, what limitations are associated with its use, and the extent to which it can be used to support patients, providers, or both. The objective of this review is to conduct a systematic evaluation of the literature for the feasibility and acceptability of using Google Glass in surgical settings and to assess the potential benefits as well as limitations of its application.

## Methods

We performed our systematic review and reporting of evidence in accordance with the Preferred Reporting Items for Systematic Reviews and Meta-Analyses guidelines (Multimedia Appendix 1) [11].

### Article Retrieval

A librarian collaboratively developed the search strategies with the senior author (SB) and ran searches in the following databases in April 2017: PubMed MEDLINE, Embase, Cochrane Central Register of Controlled Trials on the Wiley platform, Cumulative Index to Nursing and Allied Health Literature, PsycINFO (EBSCO), and IEEE Xplore. Search strategies for all databases were adapted from the PubMed MEDLINE strategy. Searches were conducted in all databases back to 2013, which is the year that Google Glass was first released. No language limits were applied. The search strategy specified keywords related to Google Glass (see Multimedia Appendix 2 for complete search strategies in each database). We also conducted a hand search for additional related articles in the *Journal of Medical Internet Research* and by searching the reference lists of key studies and relevant systematic reviews.

### Study Selection

The inclusion criteria required (1) original research articles; (2) studies that were randomized controlled trials, quasi-experimental studies, or pilot/feasibility studies; (3) Google Glass interventions; (4) studies conducted under surgical settings (preoperative, intraoperative, and postoperative); and (5) studies in clinical settings (real time or simulated). We categorized articles based on different stages or settings related to the surgical process, including the time spent preparing for surgery (preoperative setting), time spent during surgery (intraoperative setting), and time spent recovering from surgery (postoperative settings). The exclusion criteria were applied for (1) studies using technology-based interventions other than Google Glass; (2) nonsurgical setting studies; and (3) articles with more technical description of Google Glass but no clinical, usability, feasibility, and/or acceptability outcomes.

### Data Extraction and Analysis

We utilized a standardized form for data extraction that included the following items: authors’ names, publication year, country in which the study was performed, surgical application of the study, purpose of the study, description of Google Glass as a surgical intervention, participant demographics (age and sex when available), sample size, study design, results, limitations, and other study considerations. Two authors (NW and AM) screened all articles individually. Discrepancies were resolved through discussion with the senior author (SB) whenever necessary. Data were analyzed quantitatively and qualitatively.

## Results

### Literature Search

**Figure 1:**
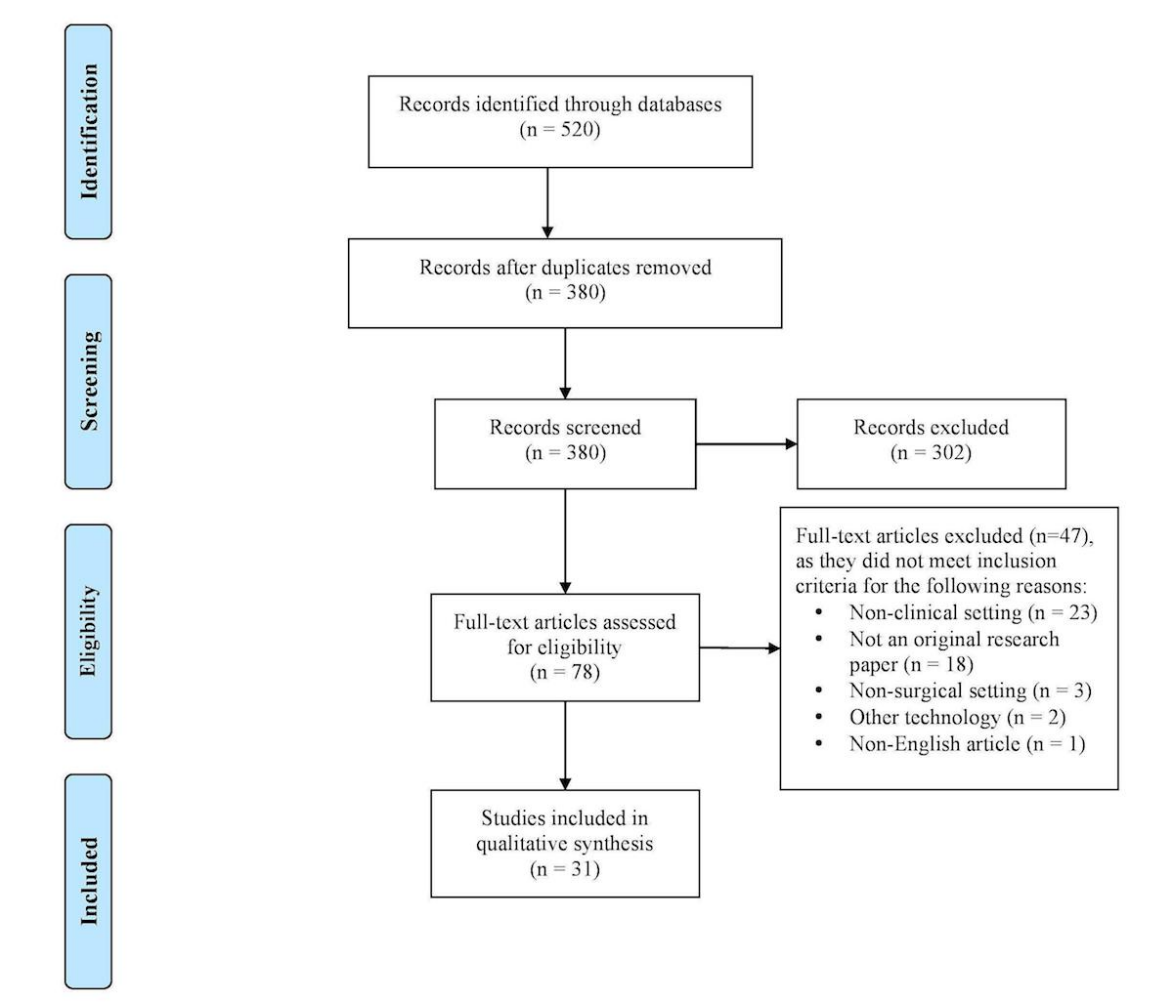
Flow of studies through the review according to PRISMA guidelines.

A total of 520 citations were retrieved through a literature search in five different databases. After removing duplicates, 380 original articles remained for screening (Figure 1). Two authors (NW and AM) independently screened the article titles and abstracts of 380 records against the inclusion criteria and a total of 78 records met all predefined inclusion criteria. Two authors (NW and AM) then independently reviewed the full text of these articles against the exclusion criteria, and 47 articles were excluded. A total of 31 articles met all predefined criteria to be included in this review. We did not identify any non-English articles that met our predefined criteria. The study flowchart and reasons for exclusion of full-text articles were documented and summarized in an adapted PRISMA study flowchart (Figure 1).

### Description of Included Studies

A summary of the 31 included studies and their Google Glass applications can be found in Table 1. Of the 31 studies, 23 (74%) were conducted in the United States [12-34], three in the United Kingdom (10%) [35-37], and one in each of Spain (3%) [38], Canada (3%) [39], Switzerland (3%) [40], China (3%) [41], Australia (3%) [42], Mongolia (3%) [19], and Brazil and Paraguay (3%) [18]. Of note, two studies from developing countries were in collaboration with researchers from the United States [18,19]. All included studies were conducted in hospital settings; 29 (94%) in adult hospitals [12-26,28-41] and two (7%) in children’s hospitals [27,42]. Sample sizes of participants who wore Google Glass ranged from N=1 to N=40. In all, 25 of 31 studies (81%) were conducted under real-time conditions or actual clinical care settings [12,14-16,18-20,23-38,40,42], whereas the other six (19%) were conducted under simulated environments [13,17,21,22,39,41]. In addition, 26 studies (84%) were pilot or feasibility studies [12,13,16-21,23,25,26,28-42], three (10%) were case studies [14,15,27], and two (6%) were randomized controlled trials [22,24].

**Table 1:** Summary of the included studies evaluating the application of Google Glass to surgical medical interventions.

Source (country)	Health condition^a^	Study design	Study setting	Google Glass application^b^
Borgmann et al, 2016 (Spain) [38]	Urology	Pilot/feasibility study	Operative	Used to record first-person point-of-view video and photos and as search engine
Iqbal et al, 2016 (United Kingdom) [35]	Urology	Pilot/feasibility study	Operative	Acted as a heads-up vital sign monitor during surgery to maintain attentiveness to surgical field
Dickey et al, 2016 (United States) [12]	Urology	Pilot/feasibility study	Operative	Served as a surgical training tool in real-time first-person visualization of urologic surgery demonstration
Chimenti & Mitten, 2015 (United States) [13]	Orthopedics	Pilot/feasibility study	Operative (simulated)	Enhanced fluoroscopic visualization of the operative field
Ponce et al, 2014 (United States) [14]	Orthopedics	Case study	Operative	Used in conjunction with the VIPAAR system to livestream video during surgery and facilitate remote telementoring between 2 surgeons, allowing real-time guidance of the operating surgeon
Armstrong et al, 2014 (United States) [15]	Orthopedics	Case study	Operative & postoperative	Facilitated medical documentation and education via video recording
Hashimoto et al, 2016 (United States) [16]	General surgery	Pilot/feasibility study	Operative	Head-mounted display allowed first-person point-of-view video recording in open surgery where placement of external cameras would be otherwise difficult; aided telementoring
Brewer et al, 2016 (United States) [17]	General surgery	Pilot/feasibility study	Operative (simulated)	Livestreamed a surgery between teacher and learner, allowing the teacher to visualize the learner’s operative field in real time and provide guidance as needed; facilitated surgical education and telementoring
Stewart & Billinghurst, 2016 (Canada) [39]	General surgery	Pilot/feasibility study	Operative (simulated)	Worn as a surgical navigation tool to help surgeon maintain attentiveness to the operative field
Datta et al, 2015 (Brazil, Paraguay, United States) [18]	General surgery	Pilot/feasibility study	Operative	Used in telementoring and improved access to quality care and education of health care providers in resource-deficient countries
Duong et al, 2015 (United States) [32]	Cardiology	Pilot/feasibility study	Preoperative	Used as a hands-free camera to help increase the accuracy of coronary angiogram interpretation
Schaer et al, 2015 (Switzerland) [40]	Cardiology	Pilot/feasibility study	Operative	Acted as a vital sign monitor; more efficient method of monitoring
Golab et al, 2016 (United Kingdom) [36]	Neurosurgery	Pilot/feasibility study	Operative	Served as an intraoperative monitoring display to decrease need for attention diversion; hands-free capabilities promoted sterility
Nakhla et al, 2017 (United States & Mongolia) [19]	Neurosurgery	Pilot/feasibility study	Preoperative, operative, & postoperative	Livestream abilities allowed students to visualize surgery in real time
Yoon et al, 2016 (United States) [20]	Neurosurgery	Pilot/feasibility study	Operative	Served as a heads-up neuronavigation monitor in pedicle screw placement; also projected video stream from external video-capture device for surgeon to view
Evans et al, 2016 (United States) [21]	Minimally invasive procedure—CVC insertion	Pilot/feasibility study	Operative (simulated)	First-person videography used to capture simulated internal jugular catheter insertions; potential to further medical education
Knight et al, 2015 (United Kingdom) [37]	Minimally invasive procedure—injectable ILR	Pilot/feasibility study	Operative	Live-broadcasted surgeries to trainees to further medical education
Liebert et al, 2016 (United States) [22]	Minimally invasive procedures—bronchoscopy & thoracostomy tube placement	Randomized controlled pilot study	Operative (simulated)	Acted as a continuous vital sign monitor to promote attentiveness and patient safety
Spencer et al, 2014 (United States) [23]	Minimally invasive procedure—tracheal intubation	Pilot/feasibility study	Operative	Helped document airway management procedures using built-in camera
Wu et al, 2014 (United States) [24]	Minimally invasive procedure—ultrasound-guided central line placement	Randomized controlled pilot study	Operative	Served as an ultrasound monitor to decrease surgeon’s need to redirect vision between operative field and traditional monitor
Vorraber et al, 2014 (United States) [25]	Minimally invasive procedure—percutaneous transluminal angioplasty	Pilot/feasibility study	Operative	Integrated and projected vital sign data to reduce need for multiple monitors in the operating room; allowed for increased attention to patient
Kantor, 2015 (United States) [26]	Surgical oncology	Pilot/feasibility study	Operative	Recorded photographs of Mohs surgery and gross Mohs sections; aided upload of electronic medical records
Zhang et al, 2016 (China) [41]	Surgical oncology	Pilot/feasibility study	Operative (simulated)	Acted as an ultrasound monitor to offer surgeon real-time feedback about the procedure without need to divert attention from operative field; smaller, more cost-effective alternative to near-infrared fluorescence imaging systems
Muensterer et al, 2014 (United States) [27]	Pediatric surgery	Case study	Preoperative, operative, & postoperative	Established Google+ hangout to permit teleconferencing
Drake-Brockman et al, 2016 (Australia) [42]	Pediatric anesthesiology	Pilot/feasibility study	Operative	Continuously monitored patient’s vital signs to decrease need for a separate monitor
Moshtaghi et al, 2015 (United States) [28]	Otolaryngology	Pilot/feasibility study	Operative	Audiovisual capabilities and Internet interface allowed hands-free commands and greater communication
Rahimy & Garg, 2015 (United States) [29]	Ophthalmology	Pilot/feasibility study	Operative	Recorded steps of scleral buckling procedure to be later used for medical education
Sinkin et al, 2016 (United States) [30]	Plastic surgery	Pilot/feasibility study	Operative & postoperative	Promoted sterility in the operating room through hands-free commands and intraoperative photography
Aldaz et al, 2015 (United States) [34]	Chronic wound care	Pilot/feasibility study	Postoperative	Allowed for more hygienic examination and photography of chronic wounds; connected to the Internet to decrease image upload time; reduced administrative errors via hands-free audiovisual recording of note dictation and patient barcodes
Baldwin et al, 2016 (United States) [31]	Organ transplant surgery	Pilot/feasibility study	Operative	Hands-free real-time video allowed quality assurance and collaboration between transplant staff and home surgeons during time-sensitive event
Gupta et al, 2016 (United States) [33]	Emergency medicine surgical consultations	Pilot/feasibility study	Preoperative	Provided near-real-time video used for surgical consultations

^a^CVC: central venous catheter; ILR: implantable loop recorder.

^b^Medical professionals wore Google Glass in all cases. VIPAAR: Virtual Interactive Presence and Augmented Reality.

**Table 2:** Summary of the study purposes and proposed Google Glass intervention methodology.

Source	Purpose^a^	Intervention^a^
Borgmann et al, 2016 (urology) [38]	To determine the feasibility, safety and usefulness of GG in urological surgery.	Participating surgeons given free rein to use GG’s features during surgery, such as taking videos and photographs, reviewing patient EMR and laboratory images, and accessing the Internet; patients were checked for postoperative complications to assess safety of GG use
Iqbal et al, 2016 (urology) [35]	To assess the feasibility of using GG as a vital sign monitor during surgery, specifically prostatectomy	GG has potential to decrease reaction time to abnormal patient vitals during surgery; participants performed a prostatectomy on a GreenLight Simulator using a standard vital signs monitor for 20 min and then using GG for 20 min; effectiveness of GG determined by the time taken to respond to abnormal vital signs, and patient blood loss and injuries
Dickey et al, 2016 (urology) [12]	To determine the feasibility of using GG for open urologic surgery as both a surgical assistant and a surgical training tool during the placement of an IPP	Trainees first shown a directional video on the IPP procedure projected onto the live view of the patient through GG; as trainees performed the IPP procedure, live footage of the OR was streamed to a remote physician through GG’s camera feature; the attending physician could provide guidance to the trainee; participants completed postoperative survey on GG
Chimenti & Mitten, 2015 (orthopedics) [13]	To assess the effectiveness of GG as an alternative to standard fluoroscopic techniques in hand surgery	Metacarpal and phalangeal fractures require Kirschner wires to be placed percutaneously with the help of fluoroscopic imaging on an external monitor; GG’s heads-up display used to visualize fluoroscopic imaging without diverting attention from the patient’s hand
Ponce et al, 2014 (orthopedics) [14]	To test the integration of GG with the VIPAAR system and evaluate the extent to which it affects remote communication and guidance between medical professionals	VIPAAR system was integrated with GG to allow a collaborator to remotely view the surgical field of the operating surgeon and virtually insert his or her hands in the surgical field to offer guidance; 2 orthopedic surgeons wore GG; surgeon A performed the shoulder arthroplasty while streaming live video to surgeon B, who was able to provide remote assistance
Armstrong et al, 2014 (orthopedics) [15]	To assess the use of GG in affecting communication, documentation, and consultation among clinicians during the care of a high-risk extremity	GG facilitated Google Hangout between operating surgeon and fellow colleagues intraoperatively; followed 1 surgeon through an intraoperative case & follow-up clinic with 1 patient; used GG to screen share between senior surgeon and junior resident to assess application to medical education
Hashimoto et al, 2016 (general surgery) [16]	To test the safety of GG use in surgery by analyzing the quality of a telementoring video recording of a Whipple procedure	Surgeons were blinded and shown video of the procedure recorded by GG vs iPhone 5; they were then asked to evaluate the video quality
Brewer et al, 2016 (general surgery) [17]	To study GG’s effect on real-time visualization of the trainee’s viewpoint by the instructor to enhance surgical education	Measured TTC completion of needle placement when operative field (quadrants) could be visualized by trainer and trainee vs TTC when trainer could no longer see operative field; 5 needles placed per quadrant
Stewart & Billinghurst, 2016 (general surgery) [39]	To determine whether GG can improve attentiveness to the surgical field by directly displaying surgical navigation information.	GG compared to (1) computer monitor and (2) wearable “through-the-lens” display in a simulated surgical task of positioning and orienting a tool on a plastic distal femur; subcondition: test dominant eye vs nondominant eye; to measure attentiveness in either case, response times were measured in response to LED illumination
Datta et al, 2015 (general surgery) [18]	To evaluate the usefulness of GG in surgical telementoring of hernia surgery	HRFU volunteer surgeons from Germany, Brazil, and US first trained 1 local surgeon each in Paraguay and Brazil by demonstrating the Lichtenstein hernioplasty in person; the local surgeons then performed the procedure while wearing GG, allowing the instructors to view a livestream of the surgery and to provide guidance as necessary
Duong et al, 2015 (cardiology) [32]	To assess the accuracy of interpretation of coronary angiograms recorded using GG	GG was used to record 15 coronary angiograms containing 17 critical findings; participants reviewed GG recordings on an iPad and a computer and compared them to the original angiograms on a desktop; participants were given 1 point for each angiogram in which they were able to determine the correct finding (17=max score); a follow-up satisfaction survey was given to evaluate participants’ satisfaction with GG image quality and ability to give recommendations based on GG videos
Schaer et al, 2015 (cardiology) [40]	To determine whether GG could be used as an ECG monitor and decrease the need for surgeons to divert attention from the operative field	Experimenters simulated 210 ECG rhythms that reflected conditions requiring immediate medical attention; participants asked to identify these issues in as little time as possible & received 1 point for a correct answer; experimental condition: ECG rhythms and heart rate displayed on GG; control condition: ECG and heart rate information displayed on a monitor screen
Golab et al, 2016 (neurosurgery) [36]	To enhance the efficiency of spinal surgery, specifically SDR, using GG	SDR procedure: identify and cut the most responsive nerves, determined by using a probe to send a current through them, producing EMG waveform data; during procedure, the neurosurgeon must often obtain a second opinion from a neurophysiologist across the OR to determine which sensory nerves to sever; GG would help maintain sustained concentration by allowing remote communication; SDR also requires reading EMG data, which would be more efficient if the probe could be integrated with GG
Nakhla et al, 2017 (neurosurgery) [19]	To test GG’s overall ease of use and effectiveness in hands-free video and photograph capture, consolidating and displaying information, and facilitating communication between medical professionals	(1) Case 1 (preoperative): GG used by attending to show residents how to prepare for a minimally invasive lumbar discectomy; GG allows hands-free commands and ability to save videos for future use; (2) case 2 (intraoperative): GG used by attending as he demonstrates the steps of a craniotomy; (3) case 3 (postoperative): GG used to record patients’ postoperative recovery during a surgical mission to Mongolia
Yoon et al, 2016 (neurosurgery) [20]	To assess the safety and feasibility of capturing and streaming neuronavigation images onto GG during spine instrumentation	Video-capture device receives signal from medical imaging device and compresses it to make it compatible with GG; video is streamed on GG screen for the surgeon to watch; measured time it took doctors to place pedicle screws on a spine; control: placed screws using standard image guidance techniques; experimental: placed screws using GG
Evans et al, 2016 (minimally invasive procedures) [21]	To compare first-person video capabilities of GG to traditional third-person techniques	Videos of a simulated CVC internal jugular catheter insertion were taken from first-person perspective using GG and third-person perspective using an observer’s head-mounted camera; videos were compared by 3 expert doctors based on 3 methods: 1 checklist and 2 global rating scales (additive and summative)
Knight et al, 2015 (minimally invasive procedures) [37]	To assess GG’s ability to stream video to a smartphone and to explore telementoring capabilities	GG was used to broadcast livestream of injectable ILR, LINQ implantation in a 20-year old woman presenting with presyncope-associated palpitations
Liebert et al, 2016 (minimally invasive procedures) [22]	To assess the feasibility of GG for real-time wireless vital sign monitoring during surgery	Control group used a standard bedside digital monitor; experimental group tested GG in combination with a standard vital sign monitor; 2 scenarios: thoracostomy tube placement and bronchoscopy; all subjects from one group switched to the other for the second scenario to test the other technique
Spencer et al, 2014 (minimally invasive procedures) [23]	To explore whether GG could be effective in recording airway management to improve education demonstrations	GG recorded airway assessment and tracheal intubation of a patient with a malocclusion of the mandible; also recorded a direct laryngoscopy of another patient
Wu et al, 2014 (minimally invasive procedures) [24]	To determine whether medical practitioners at various levels of training could use GG to perform an ultrasound-guided procedure	Experimental group: used GG to perform an ultrasound-guided central line; control group: used traditional ultrasound machine during the procedure; video recordings of practitioners’ eye and hand movements were analyzed to assess distractibility
Vorraber et al, 2014 (minimally invasive procedures) [25]	To test whether GG can enhance clinical care by providing doctors with vital sign monitoring information continuously and directly within their field of view during various procedures	Physicians used GG as vital sign monitor to perform a percutaneous transluminal angioplasty in 3 patients; participants were interviewed before and after the procedure
Kantor, 2015 (surgical oncology) [26]	To assess the use of GG in Mohs surgery and cutaneous reconstruction	120 Mohs surgery patients were evaluated by physicians wearing GG; patient medical records and history were obtained using GG; calculated rate of patient acceptance of GG
Zhang et al, 2016 (surgical oncology) [41]	To develop and test a GG system to integrate fluorescence and ultrasound image acquisition to determine sites of near-infrared emitting optical agent uptake	GG used in combination with a camera for fluorescence imaging, 12 LEDs, and an M5 ultrasound probe; phantom was created as a simulation to test feasibility of GG system; GG used to detect fluorescent ICG uptake by lymph nodes; first site where this occurs is the SLN, which normally indicates tumor site; 30 core needle biopsies conducted on the phantom; done to test accuracy of GG’s fluorescence/ ultrasound imaging in isolating tumor site under 3 scenarios: (1) GG with dual-mode (fluorescence and ultrasound) imaging, (2) GG with fluorescence imaging alone, and (3) no GG; tested GG’s dual-mode fluorescence & ultrasound-guided detection of SLN, core needle biopsy, and SLN excision in an ex vivo breast resection specimen
Muensterer et al, 2014 (pediatric surgery) [27]	To explore potential uses for GG in surgical environments and assess the quality of its functions (eg, Web searches, videoconferencing)	GG worn daily for 4 consecutive weeks by one of research study authors; a diary was kept on all pros, cons, and observations; evaluated the ergonomics, battery life, audiovisual quality, functionality, lag time, connectivity, applications, acceptance, and data privacy issues associated with GG
Drake-Brockman et al, 2016 (pediatric anesthesiology) [42]	To assess the effectiveness of GG as a patient monitoring device in a pediatric anesthetic setting	Developed a program for GG consisting of 3 parts: (1) AnaeVis: runs on GG to display patient vitals, (2) AnaeHQ: runs on laptop to collect information from patient monitoring devices, and (3) AnaeComm: allows integration of computer and GG; anesthesiologist wore GG in the OR and answered follow-up survey
Moshtaghi et al, 2015 (otolaryngology) [28]	To explore the use of GG in otolaryngologic surgery and its role in surgical education and communication	A neurotologist, head and neck surgeon, and a general otolaryngologist used GG in various otolaryngologic procedures; GG also used to communicate to another remote physician for consultation during the surgery; used program, Pristine, in conjunction with GG to stream video of the surgery to a pathologist and aid in a margin analysis
Rahimy & Garg, 2015 (ophthalmology) [29]	To assess the intraoperative use of GG in scleral buckling surgery	GG recorded several steps of scleral buckling surgery
Sinkin et al, 2016 (plastic surgery) [30]	To assess the comfort of GG use during plastic surgery, level of gaze diversion from the operative field, and quality of intraoperative photography	Residents and surgeons used GG over a 7-month period, taking pictures and videos intraoperatively using voice and wink commands; videos and photos were downloaded and reviewed postoperatively; surveys conducted to assess comfort, ease of use, and quality of images
Aldaz et al, 2015 (chronic wound care) [34]	To compare the effectiveness of GG running on the SnapCap app vs iPhone using Epic Haiku in image capture	Part 1a: GG SnapCap vs iPhone-based Epic Haiku apps and took pictures of wound on a mannequin for comparison; Part 1b: follow-up questionnaire on nurse’s preferences for (1) current SnapCap system features, (2) app preferences for SnapCap vs Epic Haiku, and (3) for the preference for future SnapCap features; Part 2: examined preference for GG’s speech-to-text wound annotation
Baldwin et al, 2016 (organ transplant surgery) [31]	To test GG in a donor organ harvest	Examined GG in live collaboration between an organ retrieval team and home surgeons to assess GG’s ability to stream intraoperative video of the organ harvest
Gupta et al, 2016 (emergency department-surgical consultations) [33]	To assess GG’s asynchronous, near-real-time recording, uploading, and viewing of visual media capabilities in facilitating remote surgical consults from the emergency department	4 physician assistants assessed patients by photographing significant findings and recording videos and laboratory imaging results using GG; images were then uploaded to a secure server and accessed remotely by a surgeon; surgeon was then able to utilize the data to determine wither changes to the existing clinical management were necessary; changes in surgeon’s confidence post GG assessment about the management plan were also evaluated through a questionnaire

^a^CVC: central venous catheter; ECG: electrocardiogram; EMG: electromyography; EMR: electronic medical record; GG: Google Glass; HRFU: Hernia Repair for the Underserved; ICG: indocyanine green; ILR: implantable loop recorder; IPP: inflatable penile prosthesis; OR: operating room; SDR: selective dorsal rhizotomy; SLN=sentinel lymph node; TTC: time-to-task completion; VIPAAR: Virtual Interactive Presence and Augmented Reality.

The vast majority of the studies examined the potential use of Google Glass as an intraoperative intervention (27/31, 87%) [12-31,35-42], whereas others observed its potential use in preoperative (4/31, 13%) [19,27,32,33] and postoperative (5/31, 16%) [15,19,27,30,34] settings. Only a few studies evaluated the use of Google Glass in more than one of these settings (4/31, 13%) [15,19,27,30]. In many cases, multiple functions and applications of Google Glass were tested in a single study. Of the two involving pediatric patients, one study required consent given by the patients’ parents or guardians, and all recordings were shared with them as requested [27]. In the other, Google Glass was not connected to the hospital network or Internet, and no recordings were made [42].

### Provider Characteristics of the Included Studies

In all studies, Google Glass was worn exclusively by a medical professional, including nurses, physician assistants, medical school students, medical residents (postgraduate years 1 to 5), attendings, or simulated health care professionals. Reporting of provider demographics varied across all studies. Three studies reported age data of health care professional participants: one reported a range of 27 to 31 years [40], one reported a mean of 29.7 years [22], and one reported a mean age of 28.4 years with a range of 18 to 50 years [39]. Two studies reported health care professional sex information: one study had a participant pool that was 14.3% (1/7) female and 85.7% (6/7) male [40] and the other reported a sample that was 100% (12/12) male [39].

### Patient Characteristics of the Included Studies

Reporting of patient demographics was largely limited across all studies. Only two studies provided patient age data: one included a sample of participants with a mean age of 70.6 years [26] and the other was a case report of a patient who was 66 years [14]. Two studies reported patient sex information: one reported a participant sample that was 58% (69/120) male [26] and the other reported one male patient (1/1) [14]. None of the studies reported race or ethnicity information.

### Description of Google Glass Use

Table 2 summarizes the goals and intervention details of each study. Six studies utilized Google Glass’s heads-up display as a vital sign monitor to facilitate improved patient monitoring and maintain attentiveness to the surgical field (6/31, 19%) [22,25,35,40-42]. Five studies (5/31, 16%) used Google Glass as a surgical navigation display to visualize ultrasound and fluorescence imaging data (3/5, 60%) [13,24,41], to visualize electromyography data (1/5, 20%) [36], and to position placement of tools on the body (1/5, 20%) [39]. Five studies used Google Glass as a videoconferencing tool to communicate with remote surgeons intraoperatively (5/31, 16%) [15,27,28,31,36]. Twenty-one studies (21/31, 68%) used Google Glass as a videography and photography device to document surgeries, laboratory images, or patient electronic medical records (7/21, 33%) [21,26,29,30,32,34,38], to assist in telementoring (4/21, 19%) [14,16-18], to document patient consultations (2/21, 10%) [19,33], to broadcast live streams (2/21, 10%) [31,37], and to enhance surgical education (7/21, 33%) [12,15,17,19-21,23]. One study used Google Glass as a hands-free search engine in the operating room (1/31, 3%) [27].

### Google Glass Utilization in Different Surgical Settings

In preoperative settings (4/31, 13%), Google Glass was used in cardiac surgery (1/4, 25%) [32], neurosurgery (1/4, 25%) [19], pediatric surgery (1/4, 25%) [27], and emergency medicine (1/4, 25%) [33]. In these studies, Google Glass was tested primarily for its use in laboratory imaging interpretation and documentation (2/4, 50%) [32,33], surgical consultations (2/4, 50%) [19,33], teleconferencing (1/4, 25%) [27], and surgical education (1/4, 25%) [19].

In operative settings (27/31, 87%), Google Glass was used in various surgical specialties, including urology (3/27, 11%) [12,35,38], orthopedics (3/27, 11%) [13-15], general surgery (4/27, 15%) [16-18,39], cardiac surgery (1/27, 3.7%) [40], neurosurgery (3/27, 11%) [19,20,36], minimally invasive surgical procedures (6/27, 22%) [21-25,37], oncologic surgery (2/27, 7%) [26,41], pediatric surgery (1/27, 4%) [42], pediatric anesthesiology (1/27, 4%) [42], otolaryngology (1/27, 4%) [28], ophthalmology (1/27, 4%) [29], plastic surgery (1/27, 4%) [30], and solid organ transplant surgery (1/27, 4%) [31]. In these studies, Google Glass was utilized as a surgical education instrument (7/27, 26%) [12,15,17,19-21,23], portable surgical imaging display (5/27, 19%) [13,24,36,39,41], live stream transmitter (2/27, 7%) [31,37], vital sign monitor (6/27, 22%) [22,25,35,40-42], communication device (5/27, 19%) [15,27,28,31,36], telementoring tool (4/27, 15%) [14,16-18], audiovisual recording device to document surgeries and patient medical records (5/27, 19%) [21,26,29,30,38], and hands-free search engine (1/27, 4%) [27].

In postoperative settings (5/31, 16%), Google Glass was used in orthopedics (1/5, 20%) [15], neurosurgery (1/5, 20%) [19], pediatric surgery (1/5, 20%) [27], plastic surgery (1/5, 20%) [30], and wound care (1/5, 20%) [34]. These studies examined the utility of using Google Glass in recovery monitoring (2/5, 40%) [15,19], telemonitoring (1/5, 20%) [15], wound management (1/5, 20%) [34], video and photo review (2/5, 40%) [19,30], and administrative billing aid (1/5, 20%) [27].

### Feasibility and Acceptability of Google Glass in Surgical Settings

Most studies (20/31, 65%) conducted formal follow-up surveys with study participants to determine the feasibility and usability of Google Glass [12,16-20,22,24-27,30,32,33,35,38-40,42]. Of the 31 studies, 28 (91%) studies assessed feasibility, usability, and/or acceptability by physicians only [12-25,28-32,34-42], two by both physicians and patients (6%) [27,33], and one by patients only (3%) [26]. The two studies (7%) that reported patients’ perceptions of using Google Glass cited a generally positive response toward its use [27,33], although one group of patients reported anxiety related to being recorded without their informed consent [27]. Additional user satisfaction, feasibility and technical results can be found in Multimedia Appendix 3.

In 19 of the studies, medical professionals were satisfied with the use of Google Glass (19/31, 61%) [12,14,17,18,20,22-25,27,30,32-38,42]. Five studies did not provide quantitative ratings on Google Glass, but concluded that it was easy to use or used successfully to livestream surgery, record procedures for later use in surgical education, or communicate with colleagues remotely (5/31, 16%) [15,19,28,29,31]. One study found the peripheral display of Google Glass superior to traditional monitors but inferior to another wearable “through-the-lens” display (1/31, 3%) [39]. One study did not find a significant difference in ease of use of reading ECG rhythms on a traditional computer screen versus Google Glass (1/31, 3%) [40]. One study found that 82% of its participants viewed Glass as inferior to traditional methods, such as videography using an Apple iPhone 5 (1/31, 3%) [16]. Three studies did not provide participant-reported ratings on acceptability (3/31, 10%) [13,21,41]. In the one study evaluating solely patients’ acceptability of the device, all patients were receptive to Google Glass (1/31, 3%) [26].

Those who viewed Google Glass favorably cited its usefulness (4/19, 21%) [18,20,22,38], educational helpfulness (4/19, 21%) [12,17,35,38], ease of use (7/19, 37%) [12,14,19,22,27,38,42], comfort (4/19, 21%) [12,24,35,42], low distractibility (4/19, 21%) [17,19,22,42], ability to aid attentiveness (3/19, 16%) [22,25,35], and image quality (1/19, 5%) [27], and acknowledged their consideration for using Google Glass in the future (4/19, 21%) [12,22,35,42]. One study also found that Google Glass allowed for greater situational awareness: during a follow-up interview, one physician remotely observed a vital sign deterioration in a patient that was thought to be stable (1/31, 3%) [25].

### Limitations of Google Glass in Surgical Settings

Despite the overall promising data regarding the feasibility and the acceptability of using Google Glass in different surgical settings, several studies (17/31, 55%) have reported a number of possible limitations associated with the use of Google Glass in these settings [13,16,19,20,24,25,27-30,33-35,37,38,40,42]. One study reported that although Google Glass was a beneficial remote communication device, it was unable to capture all relevant anatomy during a certain surgery (1/31, 3%) [28]. Other sources of apprehension arose due to short battery life (8/31, 26%) [13,19,20,25,27,29,35,38]; difficulty in hands-free features, such as the head-tilt zooms and the wink feature (5/31, 16%) [19,27,30,34,42]; data privacy concerns (4/31, 13%) [25,27,33,37]; lighting issues (4/31, 13%) [19,27-29]; Web connectivity issues (2/31, 6%) [19,27]; small screen size (2/31, 6.4%) [20,40]; image quality (1/31, 3%) [16]; distractibility (1/31, 3%) [30]; time lag (2/31, 6%) [19,24]; bulkiness (1/31. 3%) [28]; volume limitations (1/31, 3%) [27]; and overheating (1/31, 3%) [25]. These limitations indicate that further modification of Google Glass Explorer’s technical hardware is necessary before the spectacles can be integrated into the surgical field.

## Discussion

### Principal Findings

As today’s technology-centered society continues to place a growing emphasis on multitasking and unfettered access to information, Google Glass and other wearable devices have attracted the attention of consumers and corporations alike. Although Google Glass Explorer Edition failed to cater to the needs of the general public, the promising, multifunctional applications of this hands-free wearable device were appealing to several stakeholders in the health care industry, including surgeons. In this systematic review, we analyzed existing clinical studies on Google Glass to assess the feasibility, acceptability, benefits, and limitations of Google Glass in surgical settings.

Considering both the proposed strengths and limitations of using Google Glass, our review of these studies suggests that Google Glass Explorer could make the greatest potential impact in settings where it has less of an impact on patient safety, such as in aiding the surgical education of medical trainees. In its Explorer form, Google Glass is still restricted by a number of technological limitations, such as inadequate battery life and display overexposure, that might make it a beneficial supplement to traditional patient monitors but less so as an independent external monitor. Similarly, Web connectivity and poor Internet connection in isolated areas of the world still pose issues for Google Glass as a long-distance telementoring tool in situations when real-time surgical decisions are needed. Based on the studies, the environment in which Google Glass seems to provide the greatest benefit at the lowest risk to the patient is surgical education. Short-distance livestreaming of surgeries by physicians to trainees provides a unique first-person vantage point of surgeries, and Google Glass’s ability to provide augmented reality guidance in simulated surgeries has the potential to aid medical students in skill acquisition and task comprehension. For example, Evans et al [21] reported a greater checklist score, denoting a higher number of procedural steps visualized by clinicians, when using the first-person perspective Google Glass compared to a third-person external monitor. Brewer et al [17] also found that, when Google Glass was used to visualize a simulated operative field between learner and trainee, the time-to-task completion of a needle placement procedure was significantly lower.

Previous related research assessed the use of both Google Glass and similar heads-up technologies in the contexts of teleconsultation, physical therapy, pain management, telementoring, videography and photography, drug delivery, and image interpretation. However, whereas recent reviews of these studies examined the use of Google Glass in addition to other wearable devices in both general medicine and surgery [43], our systematic review exclusively considers Google Glass in surgical settings alone and draws only from research conducted clinically. Although there has been one systematic review conducted on Google Glass in surgical settings in the past, our review evaluated a greater sample size of studies (N=31) to account for the growing data on the topic; the most recent review on Google Glass in surgery included 17 studies in their analysis and relied on other systematic review articles in addition to original clinical studies in their research [44]. Therefore, our systematic review contributes to the growing evidence for the utilization of Google Glass in surgical settings. Nonetheless, the authors similarly concluded that Google Glass has the potential to positively serve the health care industry, especially in patient care and medical training.

As further research on the use of the original Google Glass in professional settings has arisen, it seems that Google Glass developers have also shifted their focus of Google Glass from the consumer market to industry settings, such as health care. Despite the cessation of Google Glass Explorer Edition production in 2015, Google Glass developer, X, announced in late July of 2017 the arrival of the Google Glass Enterprise Edition [45]. This version, intended to exclusively target businesses and commercial industries, has been quietly undergoing testing with a select group of clients. Of the 33 listed, eight (25%) are health systems (CHI Health, Dignity Health, Christiana Care Health System, Eastern Maine Medical Center, Sutter Health, Trinity Health, TriHealth, Klosterfrau Healthcare Group), and some have already attested to the benefits of this updated Glass to the medical field [46]. The majority of these corporations have been utilizing Google Glass in streamlining documentation in the consultation room. Using Google Glass as a “remote scribe,” doctors at Dignity Health reported a decrease in time spent recording notes from 33% of the day to less than 10%, allowing physicians to double the time they can spend on patient interaction [47].

Although it is uncertain whether the physicians have tested Google Glass Enterprise in a specifically surgical setting, this updated technology has already addressed many of the previously cited limitations and privacy concerns of the previous Glass Explorer. These include an upgrade in camera resolution from 5 megapixels to 8 megapixels, longer battery life, faster processor, a light that signals when video recording is taking place, and faster and more secure wireless connectivity [48]. Based on our review, most of the original research conducted on Glass Explorer that viewed Google Glass as a useful tool, based on promising data, cited these as primary sources of apprehension. Health care providers may be more willing to utilize Google Glass in the workplace if the new edition of Google Glass is able to overcome these known limitations. Thus, further research will determine whether Google Glass Enterprise will be more proficient than its predecessor in surgical settings.

Our findings were also corroborated by a recently published systematic review that assessed the feasibility of Google Glass in nonsurgical settings. In their analysis, Dougherty and Badawy [49] highlighted the responses toward the technical features of Google Glass in studies spanning a broad range of medical specialties as well as patient health concerns, from weight management to developmental disorders. The authors reported that participants, in some studies, were frustrated with Google Glass’s inadequate battery life, poor camera quality, hands-free shortcut functions, and potential to infringe on patient privacy. However, although the acceptability of Google Glass was more varied across the studies they included, our review elucidated more globally positive responses to the device in surgical settings. Nonetheless, the authors similarly found that Google Glass was most well received when leveraged as a tool for enhancing medical training. In support of our findings with the value of Google Glass in training and medical education in surgical settings, of the nine studies Dougherty and Badawy reviewed regarding student training, they reported that eight studies recommended the use of Google Glass for training purposes [10,24,42,50-54].

### Strengths and Limitations

A number of strengths in our systematic review should be mentioned. First, we completed our review following established guidelines and recommendations for established systematic reviews methodology [55-57]. Second, two authors independently completed all stages of the review process. Finally, our search strategy for different databases was developed in collaboration with a librarian information specialist with more than 10 years of experience in systematic review methodology. In addition, no language restrictions were applied to minimize possible publication bias by including most relevant studies.

Potential methodological limitations in our systematic review should be discussed. First, some studies in our review included a relatively small sample size. Second, our inclusion criteria were limited to original research articles published in peer-reviewed journals, which could have led to a possible publication bias in which only positive study results are being reported and published [58]. Finally, although our literature search of five databases was comprehensive, it is possible that we could have missed a few articles related to our research question, which is also seen in other published systematic reviews [59].

### Conclusions

In conclusion, there are promising feasibility and usability data of using Google Glass in surgical settings with particular benefits for surgical education and training. Despite existing technical limitations, Google Glass was generally well received and several studies acknowledged its potential for aiding the surgical field. As Glass Explorer’s successor, Glass Enterprise, becomes more integrated in the health care industry, further research will be necessary to evaluate the efficacy of this updated technology in supporting surgeons and their patients, especially with the growing evidence to support the efficacy of technology-based interventions, although cost-effectiveness is worth further study [60-62]. In doing so, clinicians may be able to better understand the environments in which wearable devices, such as Google Glass, can be most successful and how to offer their patients the most advanced comprehensive care.

## Appendix

Multimedia Appendix 1 PRISMA checklist.

Multimedia Appendix 2 Search strategies.

Multimedia Appendix 3 Summary of feasibility and user satisfaction results.

## Abbreviations

IEEE: Institute of Electrical and Electronics Engineers
MEDLINE: Medical Literature Analysis and Retrieval System Online
PRISMA: Preferred Reporting Results of Systematic Reviews and Meta-Analyses
