# Supplementary material for: Using Google Glass in Surgical Settings: Systematic Review
Source: JMIR Mhealth Uhealth. 2018 Mar 6;6(3):e54. doi: 10.2196/mhealth.9409 (PMC5861300; doi:10.2196/mhealth.9409)
Supplement: Multimedia Appendix 2 [file mhealth_v6i3e54_app2.pdf]

Google Glass search strings

PubMed: 13 April 2017

“google glass”
= 119 results

Embase: 13 April 2017

‘google glass’
=157 results

CINAHL: 13 April 2017
“google glass”
=32

CENTRAL: 13 April 2017
“google glass”
=9 results

PsycINFO: 13 April 2017
“google glass”
=19 results

IEEE Explore: 24 December 2017
“google glass”
=184

Total: 520

Total after de-duplication: 196
