# Supplementary material for: Using Google Glass in Surgical Settings: Systematic Review
Source: JMIR Mhealth Uhealth. 2018 Mar 6;6(3):e54. doi: 10.2196/mhealth.9409 (PMC5861300; doi:10.2196/mhealth.9409)
Supplement: Multimedia Appendix 3 [file mhealth_v6i3e54_app3.pdf]

Multimedia Appendix 3. Summary of feasibility and user satisfaction results

Source	User satisfaction results
Borgmann et al, 2016<br><br>(Urologic surgery) [12]	7 urologists performed 31 procedures of varying complexity (N=7)<br><br>Median operation time was 8.5 min shorter wearing GG than without for the same surgery performed by the same surgeon<br><br>Overall usefulness ratings of GG in surgery:<br><br>• Very high: 3 surgeons (43%)<br><br>• High: 2 surgeons (29%)<br><br>• Moderate: 1 surgeon (14%)<br><br>• Low: 1 surgeon (14%)<br><br>• Usefulness (high to low)<br><br>Taking photos & recording videos for teaching and documentation<br><br>Reviewing patients' records & performing tele-consultation<br><br>Reviewing patients' images and searching the Internet for health information<br><br>Limitations: Battery life
Iqbal et al, 2016<br><br>(Urologic surgery) [13]	Participants: 24 novices, 8 intermediates, 5 experts (N=37)<br><br>84% of surgeons responded faster to abnormal vitals when using GG<br><br>Average response time:<br><br>GG: 35.5 s<br><br>Standard vital signs monitor: 51.5 s<br><br>Mean blood loss lower when using GG<br><br>Those who found GG distracting had higher loss<br><br>36 verumontanum injuries: 24 with GG, 12 using standard monitor<br><br>GG increased vital signs awareness:<br><br>• 79% of novices (19/24)<br><br>• 75% of intermediates (6/8)

	• 80% experts (4/5)<br><br>Would use GG in a future surgery:<br><br>• 71% of novices (17/24)<br><br>• 75% of intermediates (6/8)<br><br>• 100% of experts (5/5)<br><br>• 81% participants agreed GG was comfortable (30/37)<br><br>• 76% participants agreed GG allowed greater awareness of vitals than a standard monitor (28/37)<br><br>• 68% participants would use GG in this procedure again (25/37)
Dickey et al, 2016<br><br>(Urologic surgery) [14]	Participants rated statements on 10-point Likert scale (1=not at all to 10=always)<br><br>Educational usefulness: 8.6<br><br>GG & app ease of navigation: 7.6<br><br>Likeliness to use if available: 7.4<br><br>Too distracting: 4.9<br><br>Participants asked yes/no statements<br><br>Recommend to use GG in training programs: 81% yes (21/26)<br><br>GG belongs in the OR: 93% yes (26/28)<br><br>Would consider using GG in future practice: 71% yes (20/28)<br><br>GG application bettered understanding of IPP procedure: 53% yes (9/17)
Chimenti & Mitten, 2015<br><br>(Orthopedic surgery) [15]	Significant results using GG (traditional vs GG):<br><br>Shorter average time to pin each fracture (127 vs 86 s, *P*=.02)<br><br>Fewer fluoroscopic images taken on average (5.3 vs 3.2, *P*=.01)<br><br>Fewer images taken per fracture (6.4 vs 3.6, *P* <.01)<br><br>Limitations<br><br>• Short battery life; conferencing only allowed for 30-45 min<br><br>• Privacy

	• Wi-Fi availability in certain ORs
Ponce et al, 2014<br><br>(Orthopedic Surgery) [16]	Surgery was streamed successfully<br><br>Patient surgery was successful although 45 min longer than usual<br><br>GG was considered convenient and less obtrusive than conventional OR cameras used to record the surgery
Armstrong et al, 2014<br><br>(Orthopedic surgery) [17]	Case 1: OR surgeon was able to converse with surgical colleague during the surgery using GG’s hands-free Google Hangouts app<br><br>Case 2: A junior resident was able to obtain guidance from senior attending surgeon in the OR using GG’s screen share feature
Hashimoto et al, 2016<br><br>(General surgery) [18]	N=34<br><br>GG video quality rating based on 5-point Likert scale (1=bad, 5=excellent)<br><br>50% rated fair; 50% rated it poor or bad<br><br>82.4% felt GG video quality for telementoring was inferior to that of the Apple iPhone 5
Brewer et al, 2016<br><br>(General surgery) [19]	N=11 residents (7 cardiothoracic, 4 general surgery)<br><br>User satisfaction:<br><br>• 91% had a positive experience with GG (10/11)<br><br>• 73% felt that GG was not intrusive during the surgical simulation (8/11)<br><br>• 73% agree GG would be helpful for communication (8/11)<br><br>• 91% would wear GG if it were available (10/11)<br><br>• 91% agree that it would help in surgical education (10/11)
Stewart & Billinghurst, 2016<br><br>(General surgery) [20]	N=12 subjects; performed 50 tasks<br><br>Fastest response time: through-the-lens > GG > monitor<br><br>Position/orientation: monitor had significantly less position error than the through-the-lens display and less orientation error than both the wearable displays<br><br>Effect of eye choice on response time:<br><br>• GG: no significant difference in response time between the 2 eyes; response time increased by 2.2 s (10%) when the display was over the nondominant eye<br><br>• Response time significantly faster than monitor when GG worn over

	right eye regardless of dominance<br><br>User satisfaction:<br><br>• Participants preferred the through-the-lens display for ease of use, of LED detection, and of hand/eye coordination.<br><br>• For these, GG came in second and the traditional monitor was last<br><br>• No preference in tool positioning tasks
Datta et al, 2015<br><br>(General surgery) [21]	Local surgeon was evaluated based on the Lichtenstein-Specific Operative Performance Rating Scale, which used a 3- or 5-point Likert scale for 13 items during each of the 4 procedures<br><br>Brazil trainee claimed that the value of the training program was “very valuable,” generating a moderate amount of change in his routine practice, and an increase in his confidence with Lichtenstein hernioplasty<br><br>Paraguay trainee was able to perform the operation with less guidance from the teaching surgeon after visualizing the demonstration
Duong et al, 2015<br><br>(Cardiology) [40]	N=10 physicians (2 attendings and 8 cardiology fellows)<br><br>Mean score comparisons (max=17):<br><br>• GG angiogram displayed on an iPad vs original angiogram: 14.9 ± 1.1 (*P*=.06)<br><br>• GG angiogram displayed on a desktop vs original angiogram: 15.9 ± 1.1 (*P*=.43)<br><br>• GG angiogram displayed on a desktop vs GG angiogram displayed on iPad: 15.2 ± 1.8 (*P*=.51)<br><br>User satisfaction & likelihood to give recommendations using GG:<br><br>• 10% “neutral” in regard to quality and giving recommendations (1/10)<br><br>• 60% were “somewhat satisfied” and would be “somewhat comfortable” giving recommendations (6/10)<br><br>• 30% were “very satisfied” and would be “very comfortable” giving recommendations (3/10)
Schaer et al, 2015<br><br>(Cardiology) [22]	N=7 medical residents (PGY-1 to PGY-5)<br><br>Conducted 210 trials<br><br>No statistically significant difference in frequency of correct answers (*P*=.81)<br><br>Computer: 98.5% accuracy (207/210)

	GG: 99.0% accuracy (208/210)<br><br>User satisfaction:<br><br>• Some of the participants reported slight fatigue after reading ECG rhythms on GG as a result of the small screen<br><br>• Participants reported similarly low amounts of difficulty in reading ECG rhythms on the computer screen and on GG
Golab et al, 2016<br><br>(Neurosurgery) [23]	The proposed GG platform proved effective as an intraoperative electromyography monitoring display
Nakhla et al, 2017<br><br>(Neurosurgery) [24]	Preop setting: GG was used by OR attending to show residents how to prepare for a minimally invasive lumbar discectomy; hands-free capability prevented disruption in workflow<br><br>Perioperative setting: GG worn by attending while he assisted residents in performing a craniotomy<br><br>Noted some difficulty in the hands-free head-tilt zoom feature because it required moving head closer or father from the operative field<br><br>Postoperative setting: surgeon was able to record postoperative consultation and send video<br><br>Wi-Fi in the area was unreliable, thus preventing live streams<br><br>All residents believed GG was easy to use and did not impede workflow
Yoon et al, 2016<br><br>(Neurosurgery) [25]	N=10 operations; 2 surgeons rated GG experience from 1-5 after each operation (1=poor; 5=excellent):<br><br>Helpfulness:<br><br>Rated GG more highly as the trials went on; rated 4 or 5 in operations 6-10<br><br>Image quality:<br><br>• Rated 2 or 3 in the first 3 trials<br><br>• Rated 4 after operation 7<br><br>80 yes/no responses to positive/negative statements about GG recorded:<br><br>• 79% responses indicated positive experience (63/80)<br><br>• 21% responses indicated negative experience (17/80)<br><br>Usability comments:<br><br>• Image transmission speed and quality deemed satisfactory

	• Size of the GG display was small<br><br>Average screw placement time:<br><br>• GG: 4.13 min/screw<br><br>• No GG: 4.86 min/screw
Evans et al, 2016<br><br>(Minimally invasive procedure—CVC insertion) [26]	N=10 participants (only 7 videos were obtained)<br><br>CL consisted of 10 steps in the CVC procedure; received a 0 or 1 depending on if a step was completed<br><br>Total CL score calculated by adding the 10 items (10=max score)<br><br>CL higher for the 1P perspective than 3P (7.9 vs 6.86)<br><br>GRS-a was based on 7 questions, scored 0-4 or 0-5. Total GRS-a calculated by adding the 7 items (31=max score)<br><br>No significant differences<br><br>GRS-s was a single 5 point rating based on the participants' ability to complete the task<br><br>No significant differences
Knight et al, 2015<br><br>(Minimally invasive procedure—injectable ILR) [27]	Short-distance GG livestream was considered successful<br><br>GG was not bothersome and Bluetooth pairing with Samsung Note was simple<br><br>Watching the procedure's livestream from the Samsung was reported as a good experience
Liebert et al, 2016<br><br>(Minimally invasive procedures—bronchoscopy & thoracostomy tube placement) [28]	N=14 surgery residents<br><br>Thoracostomy tube placement:<br><br>• Response time to severe hypotensive vitals was 10.1 s faster in GG group than in control (31.8 s vs 41.9 s)<br><br>• 71% less time spent looking away from operative field using GG<br><br>Bronchoscopy:<br><br>• Participants noticed critical desaturation 8.8 s earlier in GG group than in control (64.6 s vs 73.4 s)<br><br>• Significantly less time spent looking away from operative field using GG than that using a traditional monitor<br><br>Follow-up survey results (N=14 participants):

	• 64% “agreed” or “strongly agreed” that Google Glass increased situational awareness<br>• 86% felt GG was helpful in vital signs monitoring<br>• 93% saw GG as easy to use in that setting<br>• 85% saw vital sign streaming on GG could help improve patient safety<br>• 86% did not feel that GG impeded task completion<br>• Most subjects did not believe GG could replace traditional monitors fully, but would still consider using it in the future
Spencer et al, 2014<br><br>(Minimally invasive procedure—tracheal intubation) [29]	Successful visualization of airway, tracheal intubation, and recording of laryngoscopy with GG
Wu et al, 2014<br><br>(Minimally invasive procedure—ultrasound-guided central line placement) [30]	N=40 participants<br><br>87% of GG group subjects felt GG was comfortable (17/20)<br><br>GG group spent greater average time to perform the procedure<br><br>Responses to: “How likely would you be to use ultrasound visualization through Google Glass as opposed to traditional ultrasound machine monitors”?<br>• 18% responded very likely<br>• 35% moderately likely<br>• 35% somewhat likely<br>• 8% not very likely<br>• 5% not at all likely<br>• 78% would be interested in future GG research in medical education
Vorraber et al, 2014<br><br>(Minimally invasive procedure—percutaneous transluminal angioplasty) [31]	GG allowed increased concentration<br><br>Physician relied exclusively on Glass and did not use backup display at all during the surgery<br><br>GG allowed multitasking through vital signs monitoring display<br><br>Greater situational awareness<br><br>Patient vitals monitoring on GG continued even after physicians finished the procedure and left the room for a follow-up interview

	The physician observed a deterioration in patient vitals that would have been otherwise unnoticed without the wearable display<br><br>Limitations<br><br>• GG Explorer Edition has short (2 h) battery life<br><br>• Excess heat production during certain tasks cause GG to shut down abruptly<br><br>• Data privacy
Kantor, 2015<br><br>(Oncologic surgery) [32]	100% of patients (N=120) were receptive to GG use
Zhang et al, 2016<br><br>(Oncologic surgery) [33]	GG's dual-mode imaging aided core needle biopsies in phantom and *ex vivo* specimen<br><br>100% biopsy success rate when GG was used with dual-mode imaging (10/10).<br><br>70% biopsy success rate when GG used fluorescence imaging only (7/10)<br><br>20% success when biopsy done without GG guidance (2/10)<br><br>GG's dual-mode imaging was able to guide ICG detection and SLN excision in ex vivo breast sample
Muensterer et al, 2014<br><br>(Pediatric Surgery) [34]	Positive results<br><br>• High video and image quality<br><br>• Able to connect with Wi-Fi and Bluetooth smoothly<br><br>• Automatic synchronization with Google cloud server via Wi-Fi<br><br>• Hands-free Google searches<br><br>• Complex medical jargon was able to be recognized at least half the time<br><br>• Google Hangout app allowed telementoring<br><br>Limitations<br><br>• Head-tilt feature caused GG to unintentionally turn on from normal head movements<br><br>• This version of GG had 8.5-10 h battery life for everyday use; continuous video recording only lasted 30-40 min before battery died

	• Volume was too low<br>• Lighting<br>• Livestream lag time and freezing<br>• Privacy issues<br>Acceptability<br>• Overall GG was well received by patients, nurses, doctors<br>• Some patients anxious that GG could be used to record them without their consent
Drake-Brockman et al, 2016<br>(Pediatric surgery & Anesthesiology) [35]	N=40<br>10 registrars with 3-4 years training<br>7 fellows in final year of training<br>23 pediatric anesthesia consultants<br>User satisfaction:<br>• 90% found GG comfortable (36/40)<br>• 86% agreed GG was easy to read (34/40)<br>• 82.5% denied GG's distractibility (33/40)<br>• 78% might use GG in the future.<br>• 58% would recommend GG.<br>• 56% would consider wearing the device in front of patients<br>• Less experienced anesthetists were more open to this (78% of registrars vs 43% of consultants)
Moshtaghi et al, 2015<br>(Otolaryngologic surgery) [36]	Surgeon wearing GG was able to intraoperatively consult a remote physician and to stream live video of surgery to a pathologist<br>Livestream and recordings of the procedure were used to teach residents<br>Limitations<br>Wearing GG in addition to other necessary head-mounted devices, such as a headlight, might be difficult<br>Certain lighting may cause image overexposure

Rahimy & Garg, 2015<br><br>(Ophthalmic surgery) [37]	GG was able to record the scleral buckling procedure steps<br><br>Image and video quality adequate enough for others to recognize steps of the surgery<br><br>Audio quality was clear<br><br>Limitations<br><br>• Bright lighting<br><br>• Absence of flash in low-lighting<br><br>• Wide-angle lens decreased size of operative field, forcing surgeon to physically move closer to the surgical site<br><br>• Battery life
Sinkin et al, 2016<br><br>(Plastic surgery) [38]	N=9 plastic surgery attendings and residents<br><br>Survey based on 5-point Likert scale (1=very poor, 5=excellent)<br><br>Ease of voice-activated image capture: 3.11<br><br>Video capture: 3.22<br><br>Ease of wink feature: 1.89<br><br>Intraoperative image review: 2.56<br><br>Image quality: 3.89<br><br>Video quality: 3.67<br><br>Comfort: 4.56<br><br>Satisfaction: 3.78<br><br>33% thought GG was distracting<br><br>Limitations: Participants sometimes had to shift attention away from operative field to take photos using GG
Aldaz et al, 2015<br><br>(Chronic wound care) [42]	N=16 nurses<br><br>Wilcoxon Signed-ranks test showed significant preference for hands-free barcode scanning for routine clinical care (P <.001, r=.71)<br><br>Nurses favored voice-based documentation capabilities of GG (P=.01, r=.52)<br><br>Nurses significantly favored GG's double-blinking shortcut for taking photos (P <.01, r=.71)

	GG's head-tilt shortcut for zooming was the least preferred hands-free interaction method by nurses (*P*=.06)<br><br>Nurses favored GG for sharing and discussing images, EMR & image retrieval, and GG's virtual ruler
Baldwin et al, 2016<br><br>(Donor organ harvest) [39]	GG facilitated collaboration between home transplant center and field team.
Gupta et al, 2016<br><br>(Emergency department—Surgical consultations) [41]	45 evaluable surgical consultations obtained<br><br>24% of cases, surgeon changed clinical management plan based on GG-captured recordings (11/45)<br><br>4.4% changed from OR to more conservative management (2/45)<br><br>6.7% changed from conservative management to OR (3/45)<br><br>Confidence in clinical plan after viewing GG media:<br><br>44% of cases, surgeon became more confident (20/45)<br><br>Median confidence increased after viewing media<br><br>44% of cases, there was no change in confidence (20/45)<br><br>11% of cases, there was a drop in confidence (5/45)<br><br>Surgeon opinion on GG and patient receptiveness:<br><br>Attending surgeon reported media was beneficial in 64% of cases (29/45)<br><br>Media found to be unhelpful in 6.7% of cases (3/45)<br><br>Out of 17 cases where surgeon predicted GG would be unhelpful, GG was found helpful in 23% of them (4/17)<br><br>Out of 28 cases where surgeon predicted GG would be helpful, 89% were found helpful (25/28)<br><br>Majority of 276 patients surveyed about GG were comfortable with their physicians using GG

GG: Google Glass; CVC: central venous catheter; HMD: head-mounted display; EMR: electronic medical record; TTC: time-to-task completion; VIPAAR: Virtual Interactive Presence and Augmented Reality; ILR: implantable loop recorder; PTA: percutaneous transluminal angioplasty; NIR: near-infrared; HRFU: Hernia Repair for the Underserved; OR: operating room,

IPP: inflatable penile prosthesis; SDR: selective dorsal rhizotomy; ICG: indocyanine green; SLN: sentinel lymph node; EMG: electromyography; ECG: electrocardiogram; CL: checklist; 1P: 1st person; 3P: 3rd person; PGY: postgraduate year
